# Plasma Gelsolin Induced Glomerular Fibrosis via the TGF-β1/Smads Signal Transduction Pathway in IgA Nephropathy

**DOI:** 10.3390/ijms18020390

**Published:** 2017-02-12

**Authors:** Lei Zhang, Changsong Han, Fei Ye, Yan He, Yinji Jin, Tianzhen Wang, Yiqi Wu, Yang Jiang, Fengmin Zhang, Xiaoming Jin

**Affiliations:** 1 Department of Pathology, Harbin Medical University, Harbin 150081, China; Zhangleiyy12@126.com (L.Z.); han050122@163.com (C.H.); yefeiboer@163.com (F.Y.); he_yan419@163.com (Y.H.); jinyinji66@126.com (Y.J.); wtzpath@163.com (T.W.); wuyiqidd@126.com (Y.W.); jiangyang1978@hotmail.com (Y.J.); 2 Department of Microbiology, Harbin Medical University, Harbin 150081, China

**Keywords:** IgA nephropathy, plasma gelsolin, glomerular fibrosis, TGF-β1/Smadssignal transduction pathway

## Abstract

Glomerular fibrosis has been shown to be closely related to the progression and prognosis of IgA nephropathy (IgAN). However, mechanism underlying IgAN glomerular fibrosis remains unclear. Recently, our study showed that plasma gelsolin (pGSN) was decreased in the serum of an IgAN mouse model and that pGSN deposition was found in the glomeruli. Another cytokine, TGF-β1, which is closely related to glomerular fibrosis, was also found to be highly expressed in the glomeruli. In the present study, we report that pGSN induces glomerular fibrosis through the TGF-β1/Smads signal transduction pathway. This is supported by the following findings: human mesangial cells (HMCs) show remarkable morphological changes and proliferation in response to co-stimulation with pGSN and polymeric IgA1 (pIgA1) from IgAN patients compared to other controls. Moreover, ELISA assays showed that more TGF-β1 secretion was found in HMCs supernatants in the co-stimulation group. Further experiments showed increased TGF-β1, Smad3, p-Smad2/3, Smad4, and collagen 1 and decreased Smad7 expression in the co-stimulation group. Our present study implied that the synergistic effect of pGSN and pIgA induced glomerular fibrosis via the TGF-β1/Smads signal transduction pathway. This might be a potential mechanism for the glomerular fibrosis observed in IgAN patients.

## 1. Introduction

IgA nephropathy, the most common form of primary glomerulonephritis worldwide, is one of the leading causes of end stage renal disease (ESRD) [1,2]. A pathological feature of IgAN is the deposition of immune complexes, predominantly immunoglobulin A, in the glomerular mesangial area. However, the specific mechanism of deposition is still unclear. A previous study showed that the type of immunoglobulin A deposited in the glomerular mesangial area is pathogenic polymeric IgA1 (pIgA1) [3], which is characterized by aberrant glycosylated IgA1. The level of pIgA1 in the sera of patients with IgA nephropathy has been shown to be associated with disease progression [4]. Human mesangial cells (HMCs) are the predominant cell type in the glomerular mesangial area, which indicates that HMCs are the first target of pIgA1 deposition. However, until now, no study has examined other endogenous or exogenous substances that have a synergistic effect with pIgA1, which can injure HMCs.

Gelsolin, which is widely distributed in organisms from lower eukaryotes to higher mammals [5], has been shown to regulate actin reorganization in a calcium-dependent manner [6]. Plasma gelsolin (pGSN) is the extracellular isoform of gelsolin and has been suggested to act as an actin-scavenging protein in the plasma. Researchers have reported that pGSN levels are subnormal in some conditions, including trauma, burns, acute liver injury, idiopathic lung injury, and bacterial and protozoalsepsis [7,8,9,10,11,12]. Osborn et al. found that pGSN is decreased in the serum of patients with rheumatoid arthritis compared to healthy patients [13]. Our previous study found that pGSN is significantly decreased in the serum of IgAN patients and animal models, and massive pGSN depositions were found in the glomeruli [14,15]. Thus, our present study focuses on the hypothesis that pGSN may have a synergistic effect with pIgA1 in promoting HMCs proliferation and TGF-β1 secretion, which are closely related to renal fibrosis.

It has been shown that IgAN development and glomerular fibrosis are closely related. Some researchers have reported a central role for the TGF-β1/Smadssignal transduction pathway in the activation of the cellular pathomechanisms that underlie the progression of glomerular fibrosis [16]. Currently, eight different Smad proteins have been found in vertebrates. As the most important signal transduction molecule downstream of TGF-β1 in cells, Smad protein transfers the TGF-β1 signal from the cell surface to the nucleus, which can regulate target gene transcription. After the combining with its receptor, TGFβ-1 can continue to bind and activate receptor-regulated Smads (R-Smads, including Smad 1, 2, 3, 5, and 8) to form a complex. Then, by combining with the common-mediator Smads (Co-Smad, smad4), a heteromultimeric complex can be formed that can enter the nucleus and regulate the transcription of target genes. Another class of Smads are inhibitory Smads (I-Smads, including Smad6 and Smad7), which can competitively combine with the activated receptor type I, preventing phosphorylation of R-Smads [17]. Our previous study showed that TGF-β1 was increased in IgAN mouse glomeruli. However, whether the up-regulation of TGF-β1 is due to pGSN deposition has not been addressed. In our present study, HMCs were induced by in vitroco-stimulation with pGSN and pIgA1, which indicated a synergistic effect of pGSN and pIgA1 in promoting glomerular fibrosis by activating the TGF-β1/Smads signal transduction pathway.

## 2. Results

### 2.1. Cell Proliferation (MTT) of HMCs Cultured with pGSN

**Figure 1:**
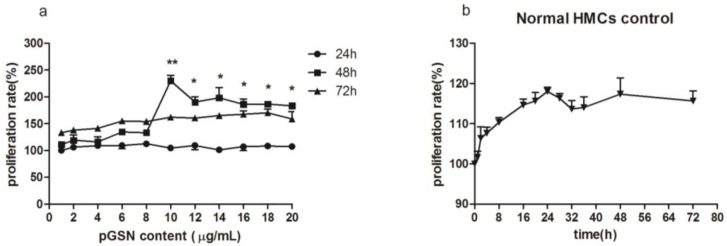
The comparison of human mesangial cells (HMCs) proliferation, which were incubated with different time and content of pGSN, and normal HMCs as control (incubated with RPMI 1640 medium). (**a**) The most significant increased proliferation rate of HMCs was observed at the point of culture with pGSN in 10 µg/mL and 48 h, when compared with any other different conditions (** *p <* 0.01). HMCs cultured with 48 handmore than 10 µg/mL pGSN had a slightly decreased proliferation rate, but still higher than other conditions (* *p <* 0.05); (**b**) Normal HMCs cultured with 1640 medium from 0 to 72 h was performed as a pGSN control, which showed no different proliferation rates at 24 and 72 h (*p* > 0.05), but significantly decreased with the 48 h group (** *p <* 0.01 with 10 µg/mL and * *p <* 0.05 with 10–20 µg/mL pGSN). Every sample in this MTT (3-(4,5-dimethylthiazol-2-yl)-2,5-diphenyltetrazolium bromide) experiment has three repeated experimental data (*n* = 3), the between-group differences were calculated with a one-way ANOVA and LSD (least-significant difference) test, and the results were presented as means ± standard deviation (SD).

We determined whether different concentrations or incubation times of pGSN produced different stimulus effects on cultured HMCs. The results showed that cell proliferation was higher in HMCs incubated with 10 µg/mL pGSN for 48 h than for other incubation concentrations or times (*p* < 0.01) (Figure 1a). Cell proliferation rate with 10 µg/mL pGSN for 48 h was higher compared with normal HMCs control (*p* < 0.01) (Figure 1b).

### 2.2. ELISA of TGF-β1 in HMCs Cultured with pGSN and/or IgA1 (from Patients or Healthy Subjects)

**Figure 2:**
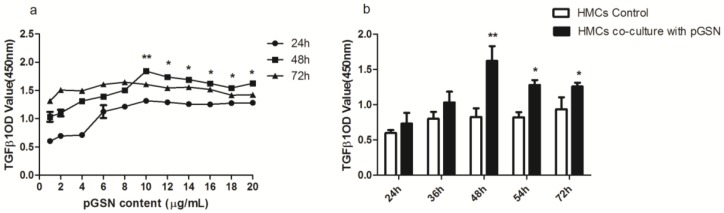
The testing result of TGF-β1 secretion by HMCs which stimulated with pGSN. (**a**) TGF-β1 had a peak secretion when HMCs were cultured with pGSN in 10 µg/mL for 48 h; (**b**) the TGF-β1 secretion by HMCs stimulated with or without pGSN, which showed that TGF-β1 secretion increased with 10 µg/mL pGSN for 36, 48, and 54 h (* *p <* 0.05), and the most obvious increased secretion was with HMCs stimulated for48 h (** *p* < 0.01). Each point in the ELISA experiment contains three repeated data (*n* = 3), the between-group differences were calculated with one-way ANOVA and LSD tests, and the results are presented as means ± standard deviation (SD).

**Figure 3:**
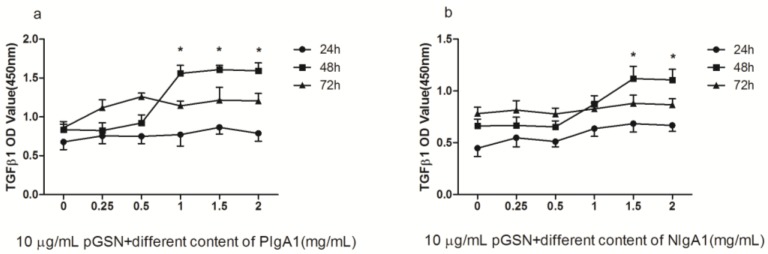
The comparison of TGF-β1 secretion by HMCs which stimulated with pGSN and different content IgA1 (PIgA1 and NIgA1). (**a**) PIgA1 treatment: the TGF-β1 secretion has a peak when HMC cultured with 10 µg/mL pGSN and 1–2 mg/mL IgA1 from IgA patients (PIgA1) in 48 h; (**b**) NIgA1 treatment: the TGF-β1 secretion has a peak when HMCs cultured with 10 µg/mL pGSN and 1.5 mg/mL IgA1 from healthy (NIgA1) in 48 h. Each sample in this experiment containsthree repeated data (*n* = 3), the between-group differences were calculated with a one-way ANOVA and LSD test, and the results are presented as means ± standard deviation (SD).

**Figure 4:**
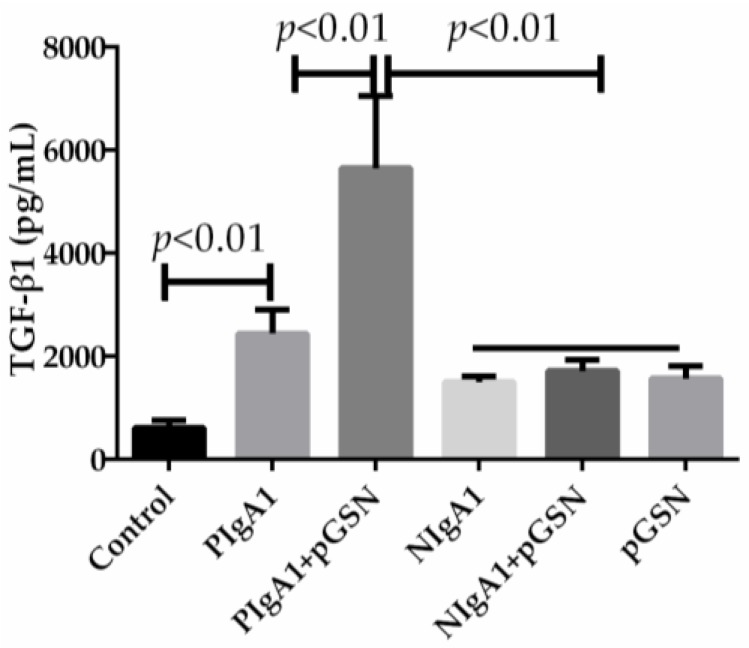
The comparison of TGF-β1 synthesis in six different HMCs treatment group. With the same content IgA1 (1.5 mg/mL), pGSN (10 µg/mL), and same time (48 h), TGF-β1 synthesis was up-regulated in any treatment group than in non-treatment group (control group). Every testing sample in this experiment has three repeated data (*n* = 3), the differences between-group were calculated with a one-way ANOVA and LSD tests, and the results are presented as means ± standard deviation (SD).

The concentrations of TGF-β1 in the pGSN and/or IgA1 (patients or healthy subjects) media were examined to determine the level of TGF-β1 synthesis by HMCs after incubation in pGSN and/or IgA1 (patients or healthy subjects) media. The synthesis of TGF-β1 was up-regulated in HMCs incubated with 10 µg/mL pGSN for 48 h compared to other concentrations and times (*p <* 0.01) (Figure 2a). The synthesis of TGF-β1 was increased in HMCs incubated with pGSN for 36, 48, and 54 h compared to corresponding HMCs cultured without pGSN (*p <* 0.01) (Figure 2b). The synthesis of TGF-β1 was up-regulated in HMCs co-incubated with 1–2 mg/mL IgA1 from patients with IgAN and 10 µg/mL pGSN compared to other concentrations (*p <* 0.01) (Figure 3a). The synthesis of TGF-β1 was up-regulated in HMCs co-incubated with 1.5–2 mg/mL IgA1 from healthy subjects and 10 µg/mL pGSN compared to other concentrations (*p <* 0.01) (Figure 3b). In addition, using the same concentration (1.5 mg/mL IgA1) and time (48 h), TGF-β1 synthesis was up-regulated in all treatment groups compared to non-treatment groups (control group) (*p <* 0.01), and TGF-β1 synthesis was up-regulated more in the pGSN + PIgA1 group than in other groups (*p <* 0.01) (Figure 4).

### 2.3. Electron Microscopy Detection

**Figure 5:**
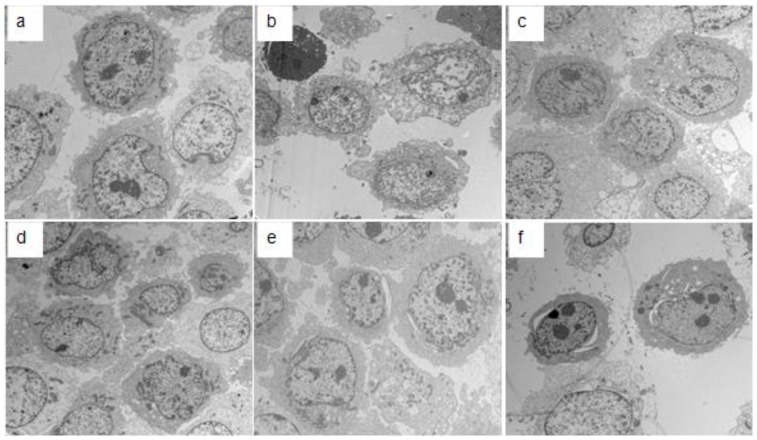
The ultrastructural changes of different stimulation group HMCs under electron microscopy. (**a**) Normal HMCs without stimulation show normal cell morphology; (**b**) HMCs under the stimulation of pIgA1 show smaller cell size, cell shrinkage, and necrosis, and organelle degeneration; (**c**) HMCs under the stimulation of pGSN + PIgA1 show that cell volume increased significantly, with HMCs having a double nucleus, nuclear fission, and cytoplasmic vacuoles; (**d**) HMCs under the stimulation of NIgA1 show individual cells show nuclear sawtooth-like change and the cytoplasm is rich in mitochondria; (**e**) HMCs under the stimulation of pGSN + NIgA1 show the increase of nucleosome and some vacuolar degeneration in the cytoplasm; and (**f**) HMCs under the stimulation of pGSN show cell shrinkage, the increase of nucleosome, and vacuolar degeneration in cytoplasm (electron microscopy, 6000×). Each group contains five samples (*n* = 5).

The ultrastructural changes of HMCs that underwent different treatments for 48 h were observed under a transmission electron microscope. As shown in Figure 5, the HMCs in the control group exhibit no notable ultrastructural changes (Figure 5a), while in the other groups, the HMCs demonstrate different degrees of pathological ultrastructural changes (Figure 5b–f). In the PIgA1 + pGSN group (Figure 5c), the HMCs display marked pathological ultrastructural changes.

### 2.4. Immunofluorescence Detection of TGF-β1 in HMCs

**Figure 6:**
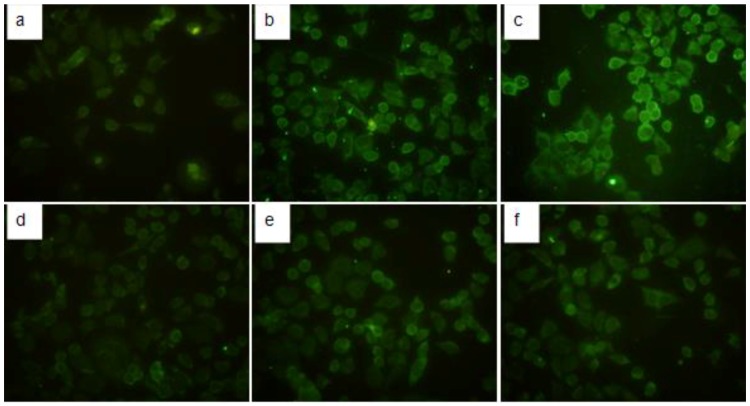
The comparison of TGF-β1 Immunofluorescence results in six different stimulation groups. (**a**) TGF-β1 expressed in normal HMCs; (**b**) TGF-β1 expressed in HMCs under the stimulation of pIgA1; (**c**) TGF-β1 expressed in HMCs under the stimulation of pGSN and PIgA1; (**d**) TGF-β1 expressed in HMCs under the stimulation of NIgA1; (**e**) TGF-β1 expressed in HMCs under the stimulation of pGSN and NIgA1;and (**f**) TGF-β1 expressed in HMCs under the stimulation of pGSN (immunofluorescence, 200×). Each group contains six samples (*n* = 6), the between-group differences were calculated with a one-way ANOVA and LSD test, the results arepresented as means ± standard deviation (SD).

The TGF-β1 expression in the six groups of HMCs with different treatment conditions (as described previously) was observed under a fluorescence microscope. As shown in Figure 6, HMCs with no treatment (Figure 6a) exhibited no obvious expression of TGF-β1. However, various degrees of immunofluorescent staining for TGF-β1 expression (green) were observed in the other groups (Figure 6b–f). In the PIgA1 + PGSN group (Figure 6c), TGF-β1 expression was notably increased compared to the other groups.

### 2.5. Western Blot Detection of TGF-β1/Smads in HMCs

**Figure 7:**
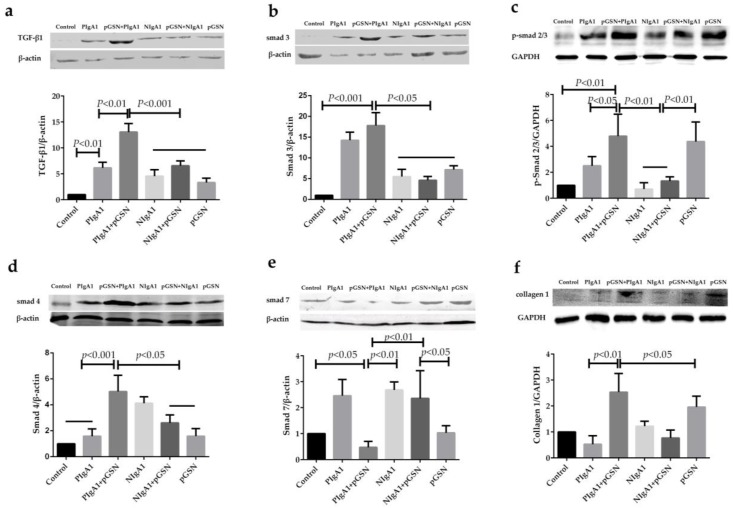
The comparison of TGF-β1, Smad3, p-Smad2/3, Smad4, Smad7, and collagen 1 expression in HMCs under different stimulation group. Comparison in six different HMCs stimulation group of (**a**) TGF-β1 expression; (**b**) Smad3 expression; (**c**) p-Smad2/3 expression; (**d**) Smad4 expression; (**e**) Smad7 expression; and (**f**) collagen 1 expression, which showed TGF-β1, Smad3, p-Smad2/3, Smad4, and collagen 1 expression have significance increased but Smad7 marked decreased in (pGSN + PIgA1) group. Each group have five test samples (*n* = 5), the between-group differences were calculated with a one-way ANOVA and LSD test, the results were presented as means ± standard deviation (SD).

The TGF-β1, Smad3, Phospho-Smad2/3 (p-Smad2/3), Smad4, Smad7, and collagen 1 protein levels of the six groups of HMCs (as described previously) were examined by Western blot analysis. As shown in Figure 7, TGF-β1, Smad3, p-Smad2/3, Smad4, and collagen 1 expression in HMCs were markedly increased in the PIgA1 + pGSN group compared to the other groups (Figure 7a–d,f), but Smad7 expression was notably decreased (Figure 7e).

## 3. Discussion

IgAN is the most common form of primary glomerulonephritis. Approximately 20%–50% of IgAN patients will develop ESRD after 10–15 years [18]. The progression of IgAN to ESRD shows a close relationship with renal fibrosis. Therefore, it is important to find an effective antagonistic target of renal fibrosis that could delay or reverse the progression of IgAN.

Gelsolin is an important cytoskeletal protein. Earlier studies have confirmed that gelsolin is involved in the pathogenesis of many diseases. Gelsolin has two subtypes, intracellular gelsolin (cGSN) and plasma gelsolin (pGSN). cGSN has been reported to play important roles in the regulation of cell apoptosis, tumor progression, phagocytosis, actin activity regulation, blood platelet formation, and cell movement [19,20,21,22,23,24,25,26,27]. pGSN plays important roles in body protection and internal environment stability as one of the most important actin-binding proteins in the actin-clearing system. Previous studies have shown that pGSN levels are closely associated with inflammatory diseases [28,29,30,31]. In addition, abnormal expression of pGSN has been reported in many types of tumors [32,33,34,35]. pGSN is decreased in many serious diseases [7,8,9,10,11,12], and a possible mechanism of this decrease is the consumption of pGSN when rapid increases in globular and filamentous actin in the circulation are cleared [11,36]. In addition, some researchers have reported that pGSN in the serum of patients with rheumatoid arthritis was significantly decreased but markedly increased in the corresponding joint fluid [13]. This finding suggests that pGSN may be involved in the pathogenesis of chronic diseases, and especially those closely related to chronic autoimmune diseases. Another recent study found that in Systemic Lupus Erythematosus (SLE) patients, pGSN serum concentrations were significantly decreased, and the disease activity index showed a significant negative correlation with pGSN serum concentrations [37]. Our previous study demonstrated that pGSN in the serum of an IgAN animal model was decreased, but deposits in the glomeruli were increased. Another finding in this study was that the expression of TGFβ1 in the glomeruli was remarkably increased. Therefore, we hypothesized that pGSN may be involved in the pathogenesis of IgAN glomerular fibrosis. To further confirm this speculation, we co-stimulated HMCs by administering pGSN protein and pathogenic serum IgA1 from IgAN patients. The MTT results showed that pGSN can stimulate the proliferation of HMCs in a dose-dependent manner. Although in our present study, HMCs proliferation with co-stimulation of pGSN and IgA1 shows no significant difference with the pGSN stimulation group. Electron microscopy results confirmed that both pGSN and Gd-IgA1 could cause varying degrees of ultrastructure changes in HMCs, and the most marked lesions were in the pGSN and Gd-IgA1 combined stimulation group. These results imply that pGSN and Gd-IgA1 have a synergistic effect in stimulating morphologic and functional changes in HMCs.

The mechanism of renal fibrosis formation is very complicated. Previous studies have proposed many different signaling pathways. However, the only widely accepted fibrogenic signal transduction pathway related to renal fibrosis is the TGFβ1/Smads signal pathway [38]. Our previous research results have shown that in an IgAN animal model, the expression of TGFβ1 was significantly increased. However, whether the increase in TGFβ1 expression was caused by stimulation of HMCs with pGSN, and whether it could activate the downstream Smads proteins that can cause renal fibrosis, were not clear. In this study, the expression and secretion of TGFβ1 by HMCs was remarkably increased in the pGSN and Gd-IgA1 co-stimulation group compared to other groups. The expression of the downstream profibrotic proteins Smad3, p-Smad 2/3, Smad4 were significantly increased, and the expression of the fibrosis competitive inhibition protein Smad7 was decreased in the pGSN and Gd-IgA1 co-stimulation group. These results suggested that in IgAN, a large number of pGSN deposits in the mesangial area become a pathogenic factor and have a synergistic effect with Gd-IgA1, which plays a key role in the morphological and functional changes in HMCs. In the present study, the organelles of the HMCs showed morphological changes, and the cells exhibited increased proliferation. Collagen 1 as fibrotic molecule of ECM and the expression and secretion of TGFβ1 of HMCs were increased after pGSN treatment. Furthermore, the TGFβ1/Smads signal transduction pathway was activated and caused renal fibrosis.

Similar to other chronic kidney diseases, IgAN disease progression can cause renal fibrosis, eventually leading to renal failure. Until now, few reports have examined the relationship between IgAN and renal fibrosis. The basic pathological feature of IgAN is the accumulation of immune complexes (immunoglobulin A mainly) in the glomerular mesangial area. However, the mechanism of immune complex deposition in the mesangial area is still unclear. Previous studies have shown that the IgA1 molecules in IgAN serum and tissues were pathogenic IgA1, which show a main feature of abnormal IgA1 glycosylation. Previous studies have proven that HMCs are the primary target of abnormal IgA1 in IgAN. However, no other endogenous or exogenous molecules that can affect HMCs have been reported. In our present study, we conducted further in vitro research based on our previous study of an IgAN animal model [39]. We concluded that pGSN and Gd-IgA1 have a synergistic effect on the mechanism of renal fibrosis in IgAN, and this effect is achieved through the TGF-β1/Smads signaling pathway.

We recognized that our study had some limitations. Although TGF-β1/Smads signal pathway has been clarified to be activated by pGSN in our present study and our group have found integrin α2/β1 may be the receptor on the HMCs [14]. However, the fibrosis caused by the TGFβ1/Smads signaling pathway may exhibit cross-talk with other signal transduction pathways. Special receptors and specific binding situation on HMCs still need to be better understood in the specific roles of pGSN in IgAN pathogenesis. The functional role of pGSN in the development and progression of IgAN will be clarified, with further studies to confirm specific binding situation of pGSN in HMCs and to explore the functional role of pGSN on IgAN fibrosis pathogenesis are warranted.

## 4. Materials and Methods

### 4.1. Materials

The reagents used for cell culture were obtained from Life Technologies (Rockville, MD, USA). Jacalinagarose was obtained from Pierce (Rockford, IL, USA). Plasma gelsolin was purchased fromcytoskeleton (Denver, CO, USA).Mouse anti-human gelsolin, mouse anti-human collagen 1 and mouse anti-human TGF-β1 were purchased from Abcam (Cambridge, MA, USA). Mouse anti-human Smad3, Smad4, Smad7, and rabbit anti-human Phospho-Smad2/Smad3 (p-Smad2/3), were purchased from Santa Cruz Biotechnology (Santa Cruz, CA, USA). FITC-conjugated rabbit anti-mouse IgG was obtained from Vector Laboratories (Burlingame, CA, USA). The TGF-β1 ELISA kit and the MTT assay kit were obtained from R & D Systems (Minneapolis, MN, USA). All other chemicals were obtained from Sigma (St. Louis, MO, USA).

### 4.2. Patients and Sera

The pooled sera of patients with IgAN were collected from the Department of Nephrology, Zhongmeng Hospital of Inner Mongolia (Hohhot, Inner Mongolia, China) from November 2011 to February 2012. For all patients, a diagnosis of IgAN was confirmed based on clinical signs and histopathological and electron microscopy findings of renal biopsies. Patients with diseases from secondary causes were excluded from the study. In addition, a control cohort of normal peripheral blood was obtained from healthy volunteers who went to the hospital for medical examinations during the same period. The study was approved by Medical Sciences Ethics Committee of Harbin Medical University and in accordance with the ethical standards of the 1975 Helsinki declaration and its later amendments ethical standards in 2008. Informed consent was obtained from all individual participants included in the study.

### 4.3. Extraction of Sera IgA1 in Patients with IgAN and Healthy Controls

IgA1 was isolated from the pooled sera of 20 patients or 15 healthy controls separately by Jacalin affinity chromatography. The procedures were performed according to Wang [40]. Briefly, the pooled sera were diluted 1:1 with phosphate-buffered saline (PBS) and centrifuged at 1500 rpm for 5 min. Then, 2 mL of supernatant was placed on the Jacalin affinity column that was prepared with Jacalin immobilized cross-linked 4% beaded agarose with an IgA1 binding capacity of 2–4 mg/mL of gel. After the pooled sera of samples were incubated on the Jacalin affinity column for 30 min, the column was then washed with 175 mMTris-HCl (pH 7.4) until the optical density (OD 280 nm) was less than 0.10. IgA1 was eluted with 0.1 Mmelibiose (Yuanye Biotechnology Limited Company in Shanghai of China) in 175 mMTris-HCI in 3.0 mL fractions until the optical density returned to 0.1. After dialysis and freezing drying of the Jacalin binding proteins (from patients with IgAN and healthy controls), 2 mg/mL samples were separated by molecular sieve chromatography using a 2.0 cm × 57 cm Sephacryl S-200 HR column on a Pharmacia Smart System (ÄKTA prime plus) equipped with a micropeak detector (Pharmacia Biotech, Uppsala, Sweden). Then, the Sephacryl S-200 HR column was eluted with buffer solution, and three distinct peaks in the OD280 absorption profile were obtained, which corresponded to poly IgA1 (pIgA1), monomeric IgA1 (mIgA1), and other non-IgA1 proteins. The mIgA1 concentrations in the samples of patients with IgAN and healthy controls were tested with a BCA (bicinchoninic acid assay) kit (BiYuntian Biotechnology Research Institute, Shanghai, China) according to the manufacturer’s instructions. The molecular size of the mIgA1 fraction was determined by SDS-PAGE and Western blot analysis using mouse anti-human IgA1 antibody. To prepare aggregated IgA1 (aIgA1), samples containing identified mIgA1 were heated at 63 °C for 150 min. The molecular size of the aIgA1 fraction was determined by SDS-PAGE, while the purity of the aIgA1 (95%) was calculated as the ratio of aIgA1 concentration over the total protein concentration using a BCA kit. The aIgA1 products were placed into an ultra-dry frozen instrument for freeze drying for another 24 h to improve their concentration for subsequent experiments.

### 4.4. Cell Culture

Human mesangial cells (HMCs) were obtained from the cell bank of the Chinese Academy of Science (Shanghai, China).The HMCs were cultured in RPMI (Roswell Park Memorial Institute) 1640 medium supplemented with 10% FBS, glutamine (2 mmol/L), penicillin (50 U/mL), and streptomycin (50 µg/mL). The cells were incubated at 37 °C in 5% CO_2_ and 95% air. Mesangial cells have a stellate appearance and grow in clumps. To determine the dose- or time-course of proliferation of HMCs and the expression or synthesis of TGF-β1 by HMCs cultured with PGSN, growth arrested HMCs were cultured with pGSN (0–20 µg/mL) for 24 to 72 h and prepared for experimental testing.

### 4.5. 3-(4,5-Dimethylthiazole-2-yl)-2,5-Diphenyl Tetrazoliumbromide (MTT) Assay

The proliferation of HMCs was determined with an MTT assay kit. Briefly, growth-arrested HMCs were seeded onto 96-well plates (1 × 10^5^ cells per well) before they were stimulated with pGSN (0–20 µg/mL) for 24, 48, and 72 h. A normal HMCs cultured with 1640 medium from 0 to 72 h was performed as a pGSN control. The MTT reagent was then added, and the cells were incubated at 37 °C until a purple precipitate was clearly visible under an inverted microscope. The detergent reagent was finally added, and the cells were incubated in the dark at 37 °C until the purple precipitate was solubilized. The absorbance was measured using 550 nm as the primary wavelength and 650 nm as the reference wavelength.

### 4.6. ELISA Detection

The amount of TGF-β1 in the supernatant of the HMCs culture was determined with an ELISA kit from R & D Systems (Minneapolis, MN, USA) according to the manufacturer’s instructions. Groups of cell supernatants were diluted (1:10).The OD values were determined at 450 nm with a microplate reader.

### 4.7. Electron Microscopy Detection

HMCs with different treatment conditions were converted into cell suspensions by trypsinization. The cells were centrifuged 10 min at 3000 rpm. After discarding the supernatant, the cells were fixed 2 h in 2.5% glutaraldehyde. After washing with PBS, the cells were fixed for 1 h in 1% osmic acid. After three washes with distilled water for 5 min each, the cells were dehydrated with an ethanol gradient. The cells were embedded with ethoxyline resin and then cut on an ultramicrotome. The ultrastructure of different groups of cells was observed with transmission electron microscopy, and images were collected.

### 4.8. Immunofluorescence Detection

Six groups of HMCs with different treatment conditions were harvested, and microscope slides were prepared. After fixation in 4% paraformaldehyde, the cells were blocked with goat serum for 30 min at room temperature. The cells were incubated in mouse anti-human TGF-β1 antibody diluted 1:50 in PBS at 4 °C for 18 h. After washing with PBS, the cells were incubated with FITC (Fluorescein Isothiocyanate) conjugated rabbit anti-mouse secondary antibody diluted 1:100 at 37 °C for 30 min. After washing with PBS and mounting with 10% glycerol, the cells were observed under a fluorescence microscope (Nikon E800, Tokyo, Japan), and images were acquired.

### 4.9. Western Blot Detection

HMCs with different treatment conditions were harvested and dissolved in protein extraction buffer containing protease inhibitor cocktails. The total protein from HMCs (80 µg) in all groups was subjected to SDS-PAGE on a 10% gel and then transferred to a nitrocellulose membrane. The membrane was incubated overnight at 4 °C with mouse TGF-β1 (1:1000), Smad3 (1:1000), Smad4 (1:500), Smad7 (1:800), Collagen 1(1:1000), Rabbit p-Smad2/3(1:1000), anti-β actin (1:1000), or GAPDH (1:2000) antibodies in PBS before incubation with the appropriate peroxidase-labeled secondary antibodies. The reaction was detected with ECL plus chemiluminescent detection reagent (Amersham Pharmacia Biotech, Uppsala, Sweden). The images were scanned, and bands were quantified by Quantity One Software v4.62 (Bio-Rad, Hercules, CA, USA).

### 4.10. Detection of the HMCs after Costimulation with pGSN and IgA1

The HMCs were divided into six different dosage groups (0, 0.25, 0.5, 1, 1.5, and 2 mg/mL of aIgA1) after synchronization with pGSN. The aIgA1 from patients was incubated with the co-cultured HMCs along with 10 µg/mL of pGSN for 48 h at 37 °C. As previously described, ELISA assays for TGF-β1 and MTT were performed to determine the optimal stimulus concentration of IgA1. However, the six groups of cells were treated differently after synchronization, and the experiments were grouped as follows: HMCs without treatment (control); HMCs + IgA1 from patients with IgAN (PIgA1); HMCs + IgA1 from healthy controls (NIgA1); HMCs + 10 µg/mL of pGSN (PGSN); HMCs + 10 µg/mL of pGSN + IgA1 from patients with IgAN (PGSN + PIgA1); and HMCs +10 µg/mL of pGSN + IgA1 from healthy controls (pGSN + NIgA1). All of the groups of cocultured cells were cultured for 48 h at 37 °C. MTT assays, ELISA assays, electron microscopy, TGF-β1 immunofluorescence and Western blot analysis for elements of the TGF-β1/Smads signal transduction pathway, including TGF-β1, Smad3, Smad4, and Smad7, were performed to clarify the synergistic effects of pGSN and IgA1.

### 4.11. Statistical Analysis

The results were analyzed with SAS software version9.1 (SAS Institute Inc., Cary, NC, USA). The between-group differences were calculated with a one-way ANOVA and least-significant difference (LSD) test. Data were expressed as mean ± standard deviation (SD), and *p* < 0.05 was considered to be statistically significant.

### 4.12. Ethics Statement

Human blood samples were obtained from IgAN patients and healthy donors after informed consent in accordance with ethical standards of the Declaration of Helsinki. The protocol of the study was approved by the Ethics Committee of Harbin Medical University (PermitNumber: HMUIRB20150025).

## 5. Conclusions

In conclusion, our present study implied that pGSN and Gd-IgA1 have a synergistic effect on the mechanism of renal fibrosis in IgAN, and this effect is achieved via the TGF-β1/Smads signaling pathway.

## References

[1] Barratt J., Feehally J. (2005). IgA nephropathy. J. Am Soc. Nephrol..

[2] Donadio J.V., Grande J.P. (2002). IgA nephropathy. N. Engl. J. Med..

[3] Jan N., Bruce A.J., Milan T., Jiri M. (2008). IgA glycosylation and IgA immune complexes in the pathogenesis of IgA Nephropathy. Semin. Nephrol..

[4] Zhao N., Hou P., Lv J.C., Zina M., Li Y.F., Krzysztof K., Ali G.G., Jan N., Zhang H. (2012). The level of galactose-deficient IgA1 in the sera of patients with IgA nephropathy is associated with disease progression. Kidney Int..

[5] McGough A.M., Staiger C.J., Min J.K., Simonetti K.D. (2003). The gelsolin family of actin regulatory proteins: Modular structures, versatile functions. FEBS Lett..

[6] Yin H.L., Stossel T.P. (1979). Control of cytoplasmic actin gel-sol transformation by gelsolin, a calcium dependent regulatory protein. Nature.

[7] Suhler E., Lin W., Yin H.L., Lee W.M. (1997). Decreased plasma gelsolin concentrations in acute liver failure, myocardial infarction, septic shock, and myonecrosis. Crit. Care Med..

[8] Mounzer K.C., Moncure M., Smith Y.R., Dinubile M.J. (1999). Relationship of admission plasma gelsolin levels to clinical outcomes in patients after major trauma. Am. J.Respir. Crit. Care Med..

[9] Di Nubile M.J., Stossel T.P., Ljunghusen O.C., Ferrara J.L., Antin J.H. (2002). Prognostic implications of declining plasma gelsolin levels after allogeneic stem cell transplantation. Blood.

[10] Christofidou-Solomidou M., Scherpereel A., Solomides C.C., Muzykantov V.R., Machtay M., Albelda S.M., DiNubile M.J. (2002). Changes in plasma gelsolin concentration during acute oxidant lung injury in mice. Lung.

[11] Rothenbach P.A., Dahl B., Schwartz J.J., O’Keefe G.E., Yamamoto M., Lee W.M., Horton J.W., Yin H.L., Turnage R.H. (2004). Recombinant plasma gelsolin infusion attenuates burn-induced pulmonary microvascular dysfunction. J. Appl. Physiol..

[12] Lee P.S., Waxman A.B., Cotich K.L., Chung S.W., Perrella M.A., Stossel T.P. (2007). Plasma gelsolin is a marker and therapeutic agent in animal sepsis. Crit. Care Med..

[13] Osborn T.M., Verdrengh M., Stossel T.P., Tarkowski A., Bokarewa M. (2008). Decreased levels of the gelsolin plasma isoform in patients with rheumatoid arthritis. Arthritis Res. Ther..

[14] Zhang L., Kong D., Meng H., Han C., Zhu J., Qiao J., He Y., Wang T., Li X., Zhang F., Jin X. (2016). PlasmaGelsolinPromotes Proliferation of Mesangial Cell in IgA Nephropathy. Cell Physiol. Biochem..

[15] Han C.S., Zhang L., Zhu X.L., Tang J., Jin X.M. (2013). Plasma gelsolin levels are decreased and correlate with fibrosis in IgA nephropathy. Exp. Biol. Med..

[16] Roberts A.B. (2002). The ever-increasing complexity of TGF-β signaling. Cytokine Growth Factor Rev..

[17] Wang W., Koka V., Lan H.Y. (2005). Transforming growth factor-β and Smadssignalling in kidney diseases. Nephrology.

[18] Magistroni R., Furci L., Leonelli M., Rosen R.M., Fein D.A. (2006). A validated model of disease progression in IgA nephropathy. J. Nephrol..

[19] Cho J.E., Park W., Kim D.C. (2012). Down-regulation of gelsolin may play a role in the progression of inverted papilloma through an antiapoptotic mechanism. Am. J. Rhinol. Allergy.

[20] Qiao H., Koya R.C., Nakagawa K. (2005). Inhibition of Alzheimer’samyloid-βpeptide-induced reduction of mitochondrial membrane potential and neurotoxicity by gelsolin. Neurobiol. Aging.

[21] Litwin M., Nowak D., Mazur A.J. (2012). Gelsolin affects the migratory ability of human colon adenocarcinoma and melanoma cells. Life Sci..

[22] An J.H., Kim J.W., Jang S.M. (2011). Gelsolin negatively regulates the activity of tumor suppressor p53 through their physical interaction in hepatocarcinoma HepG2 cells. Biochem. Biophys. Res. Commun..

[23] Tanaka H., Shirkoohi R., Nakagawa K. (2006). SiRNAgelsolin knockdown induces epithelial-mesenchymal transition with a cadherin switch in human mammary epithelial cells. Int. J. Cancer.

[24] Shao F., Zhang R., Don L., Ying K. (2011). Overexpression of gelsolin-like actin-capping protein is associated with progression of lung adenocarcinoma. Tohoku J. Exp. Med..

[25] Peng K.W., Liou Y.M. (2012). Differential role of actin-binding proteins in controlling the adipogenic differentiation of human CD105-positive Wharton’s Jelly cells. Biochim. Biophys. Acta.

[26] Silacci P., Mazzolai L., Gauci C., Stergiopulos N., Yin H.L., Hayoz D. (2004). Gelsolin superfamily proteins: Key regulators of cellular functions. Cell Mol. Life Sci..

[27] Philchenkov A.A. (2003). Caspases as regulators of apoptosis and other cell functions. Biochemistry.

[28] Di Nubile M.J. (2008). Plasma gelsolin as a biomarker of inflammation. Arthritis Res. Ther..

[29] Juusela P., Tanskanen M., Nieminen A. (2009). Hereditary gelsolin amyloidosis mimicking Sjogren’s syndrome. Clin. Rheumatol..

[30] Oikonomou N., Thanasopoulou A., Tzouvelekis A. (2009). Gelsolin expression is necessary for the development of modelled pulmonary inflammation and fibrosis. Thorax.

[31] Mateos J., Lourido L., Ferna’ndez-Puente P. (2012). Differential protein profiling of synovial fluid from rheumatoid arthritis and osteoarthritis patients using LC-MALDI TOF/TOF. J. Proteom..

[32] Tsai M.H., Wu C.C., Peng P.H. (2012). Identification of secretory gelsolin as a plasma biomarker associated with distant organ metastasis of colorectal cancer. J. Mol. Med..

[33] Pan S., Chen R., Brand R.E. (2012). Multiplex targeted proteomic assay for biomarker detection in plasma: A pancreatic cancer biomarker case study. J. Proteome Res..

[34] Jin S., Shen J.N., Peng J.Q., Wang J., Huang G., Li M.T. (2012). Increased expression of serum gelsolin in patients with osteosarcoma. Chin. Med. J..

[35] Lin C.P., Chen Y.W., Liu W.H. (2012). Proteomic identification ofplasma biomarkers in uterine leiomyoma. Mol. Biosyst..

[36] Xu J.F., Liu W.G., Dong X.Q., Yang S.B., Fan J. (2012). Change in plasma gelsolin level after traumatic brain injury. J. Trauma Acute Care Surg..

[37] Hu Y.L., Li H., Li W.H., Meng H.X., Fan Y.Z., Li W.J., Ji Y.T., Zhao H., Zhang L., Jin X.M. (2013). The value of decreased plasma gelsolinlevels in patients with systemic lupus erythematosusandrheumatoid arthritis in diagnosis and disease activity evaluation. Lupus.

[38] Bottinger E.P., Bitzer M. (2002). TGF-signaling in renal disease. J. Am. Soc. Nephrol..

[39] Zhang L., Ye F., He Y., Kong D., Han C., Zhao Z., Zhu J., Liu X., Jin X. (2010). Establishment of a mouse IgA nephropathy model with MBP-20-peptide fusion protein. Anat. Rec..

[40] Wang Y., Zhao M.H., Zhang Y.K., Li X.M., Wang H.Y. (2004). Binding capacity and pathophysiological effects of IgA1 from patients with IgA nephropathy on human glomerular mesangial cells. Clin. Exp. Immunol..

